# On effects of freezing and thawing cycles of concrete containing nano-$\mathbf {SiO_2}$: experimental study of material properties and crack simulation

**DOI:** 10.1038/s41598-023-48211-4

**Published:** 2023-12-14

**Authors:** O. Arasteh-Khoshbin, S. M. Seyedpour, M. Brodbeck, L. Lambers, T. Ricken

**Affiliations:** 1 Institute of Structural Mechanics and Dynamics, Faculty of Aerospace Engineering and Geodesy, University of Stuttgart, Stuttgart, Germany; 2 Porous Media Laboratory, Institute of Structural Mechanics and Dynamics in Aerospace Engineering, Faculty of Aerospace Engineering and Geodesy, University of Stuttgart, Pfaffenwaldring 27, 70569 Stuttgart, Germany

**Keywords:** Civil engineering, Mechanical engineering

## Abstract

Construction during cold weather can lead to freezing accidents in concrete, causing significant hidden threats to the project’s performance and safety by affecting the mechanical properties and durability reduction. This study aims to deduce the compressive strength and durability of the concrete containing nano-$\mathrm{SiO}_2$ under freezing-thawing cycles with the Caspian seawater curing condition. The specimens were subjected to freezing-thawing cycles according to ASTM C666. Furthermore, crack propagation in the concrete after freezing-thawing cycles is simulated. The results reveal that adding until nano-$\mathrm{SiO}_2$ until 6% improved compressive strength before and after freezing-thaw cycles. The water permeability experiences a substantial reduction as the amount of nano-$\mathrm{SiO}_2$ increases. Furthermore, the water permeability exhibits a positive correlation with the number of cycles, resulting in significantly higher values after 150 cycles compared to the initial sample. Moreover, adding 8% nano-$\mathrm{SiO}_2$ reduced the depth of water permeability and chloride ion penetration after 150 cycles by 57% and 86%, respectively. The crack simulation results indicate that concrete containing 6% nano-$\mathrm{SiO}_2$ shows an optimal resistance against crack formation. Concrete with 6% nano-$\mathrm{SiO}_2$ requires 13.88% less force for crack initialization after 150 freezing and thawing cycles. Among different nano-$\mathrm{SiO}_2$ percentages, 6% shows the best crack resistance and 8% the minimum water permeability and chloride ion penetration.

## Introduction

Concrete is the most extensively utilized material in ports, houses, wharves, bridges, roads^1^. It has advantages such as convenience in construction, abundant source materials, good moldability, the low price^2^, high durability, and good bonding performance with reinforcing plates and steel bars^3^. Civil concrete structures experience millions of repeated load cycles during their service life^4^. It is highly believed that the growth of microcracks and deterioration of pore structure in concrete as a result of cyclic mechanical and thermal loading accelerate the damage accumulation of concrete^5^. Furthermore, concrete is simultaneously affected by environmental factors, such as freezing and thawing, deicing salts, $\mathrm{CO}_2$, and chloride ions, in marine^6–9^. The integrated attack of environmental actions and mechanical loads results in early deterioration of concrete^10^, which can be extensively classified into two kinds of failure including drastic deterioration of the concrete’s durability (i.e. permeability) and major fatigue cracks’ unstable propagation^5^.

One factor that reduces the stability and durability of concrete is damage caused by freezing and thawing processes. Here, the hydraulic concrete structures are damaged in the cold regions, during day and night as a result of fluctuation in temperature. Freezing water increases it’s volume by 9%. This expansion causes pressure forces inside the concrete, which favors the formation of internal cracks. Water can penetrate into the concrete during the thawing procedure, through leak joints and cracks. Moreover, the penetrated water was frozen at the freezing stage, and the concrete’s strength was reduced owing to the formation of cracks. There is a close relationship between the freezing and thawing durability of concrete and its pore structure. The size distribution, volume, and radius of pores determine the pore solution’s freezing point and the quantity of ice formed in pores. Normally, within a certain temperature interval, greater internal hydraulic pressure is induced by more frozen concrete pore solution, thus, more severe frost damage^11^. This is caused by freezing and thawing cycles, loss in mass^12,13^, and changes in mechanical^14^ and dynamic properties^15,16^. One of the major causes of reduction in the concrete durability is freezing and thawing, which is caused by fluctuations in temperature^17^. Freeze and thaw resistance is often a key index for evaluating the concrete’s durability. Moreover, the relative elastic modulus is measured along with strength loss rate and mass loss rate after freezing and thawing cycles to determine the frost resistance of concrete^18,19^.

To minimize the damage caused by such processes, the composition of the concrete can be improved. For this purpose, the air-entraining agent is commonly utilized for concrete mixing design to enhance the concrete structures’ freezing-thawing resistance^15,20^. It also reduces the strength of concrete. Moreover, pozzolans can result in a very compact microstructure of concrete, thus, improving frost resistance^21^. Also, pozzolans, admixtures such as silica fume, fiber materials, fly ash, and nano-materials can enhance the durability and frost resistance of concrete and preserve its strength.

Nano-materials are ultra-fine with large surface area, particle sizes of 1-100 nm and small particle size, and higher surface energy with dimensional, volume, and surface effects^22^. Nano-materials can significantly enhance the durability of concrete and mortar^23^. Several nano-materials represent great performance for improving the features of concrete like nano-clay^24^, nano-rice husk ash^25^, carbon nanotubes^26^, nano-$\text{TiO}_2$, nano-$\text{ZnO}_2$, nano-${\text{Fe}}_2O_3$, nano-$\text{CaCO}_3$^27–33^, nano-$\text{SiO}_2$ (NS)^23^, nano-${\text{Al}}_2\mathrm{O}_3$^34^ enhancing the durability and mechanical characteristics of concrete.

NS as a white fluffy powder is made of amorphous silica powder. It represents strong surface adsorption, a large specific surface area, excellent chemical purity, large surface energy, and good dispersion as a result of its tiny particle size^35^. Since hydrophilic NS has higher dispersion in water, it is mostly used in concrete^36^. NS may act as a nano-filler, occupying the voids between calcium-silicate-hydrate (C-S-H) gel particles. Moreover, NS is a pozzolan with a higher pozzolanic reaction rate owing to its large surface area to volume ratio, which indicates higher chemical activity. Concrete’s compressive strength can be enhanced using NS^37,38^. Moreover, adding NS can improve the tensile strength of the concrete^39^. Freezing and thawing resistance of the concrete is improved significantly by incorporating an optimum amount of NS into the concrete^37^. NS reduced the capillary voids in the hardened concrete. Therefore, adding NS enhances the durability of concrete against the freezing-thawing cycles^40^.

As discussed previously, adding NS to concrete, significantly affects it’s mechanical properties and therefore the underling mechanisms, when it comes to damage. Within this context, evolving micro-cracks can lead to macroscopic failure. A mathematics description of such phenomena is challenging, as the evolution of a discontinuous displacement field has to be captured. Methods like the interface- or extended finite element^41–43^ method resolve these discontinuities explicitly, but gained insufficient results, when it comes to crack branching, and further required highly specialsed code frameworks. A more recent approach, based on a so called phase-field, regularizes these discontinuities by introduction of an energy driven field-variable, blending between intact and broken material^44^. Further developments included the more physics based formulation of the crack driving energy and efficient treatment of the irreversibility of cracks^45,46^. Even if initially formulated for fracture within brittle materials, the phase-field based fracture models have been extended to ductile materials^47^ or multi-physical problems like thermo-mechanics^48,49^ or even multi-phasic continuas such as fluid-filled, porous structures^50^.

Due to the extensive use of concrete piers, breakwater decks, and retaining walls in the construction of ports and docks, studies on the mechanical properties and durability of concrete structures cured in seawater under freezing and thawing cycles is of great importance. On the southern coast of the Caspian Sea with occasionally heavy snowfalls, the average temperature is less than 0$^\circ \hbox {C}$ for three months^51^. Therefore, there is a significant impact of the freeze and thaw environment in this region. Owing to the effects of freeze and thaw damage, countermeasures are required to avoid the deterioration of reinforced concrete structures in bridges, main construction, and tunnels. Since a few studies investigated concrete properties under freezing and thawing cycles with the curing condition of Caspian seawater, in order to address this knowledge gap, the present work examined the effects of adding different dosages of NS on the compressive strength, water permeability, chloride ion penetration, crack formation in the concrete under different freezing and thawing cycles with Caspian seawater curing conditions.

## Materials and methods

### Materials

**Figure 1:**
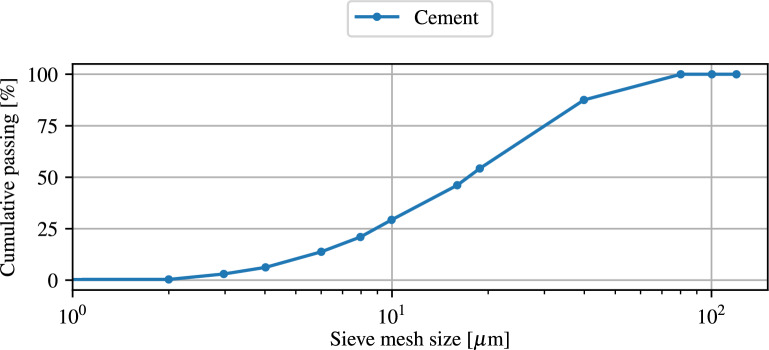
Particle size distribution curves of cement.

In this study, a type II ordinary Portland cement is utilized as the main cementitious binder in accordance with ASTM C 150^52^. The cement is produced by Khazar Cement Company (Guilan province, Iran). The fineness of cement was 3230 $\text{g}/\text{cm}^2$ in accordance with ASTM C204-11^53^ and the initial and final setting times were equal to 110 and 170 min, respectively. Also, the 7 and 28 day compressive strengths and the specific weight of cement were 415 $\text{g}/\text{cm}^2$, 535 $\text{g}/\text{cm}^2$ and 3150 $\text{g}/\text{cm}^2$, respectively. The chemical and physical properties of the used cement are given in Tables 1 and 2, respectively. The particle size distribution curve of the cement is shown in Fig. 1.

**Table 1:** The physical characteristics of cement.

Autoclave expansion $[\%]$	0.07
Specific gravity $\left[ \dfrac{\text{g}}{\text{cm}^3}\right]$	3.07
Specific surface $\left[ \dfrac{\text{cm}^2}{\text{g}}\right]$	3120
Initial setting time $[\mathrm{min}]$	140
Final setting time $[\mathrm{min}]$	190
7-day compressive strength $\left[ \dfrac{\text{N}}{\text{mm}^2}\right]$	22.1± 0.4
28-day compressive strength $\left[ \dfrac{{ \mathrm N}}{\text{mm}^2}\right]$	28.3± 0.2

**Table 2:** The chemical characteristics of cement (wt %).

$\text{CaO}$	$\mathrm {SiO_{2}}$	$\mathrm {Al_{2}O_{3}}$	$\mathrm{Fe_{2}O_{3}}$	$\mathrm {SO_{3}}$	$\text{MgO}$	Free $\text{CaO}$	Loss on ignition	$\mathrm {K_{2}O}$	Insoluble residue	$\mathrm {Na_{2}O}$
63.7	20.55	4.5	4.35	2.45	1.8	1.46	1.42	0.54	0.47	0.35

**Figure 2:**
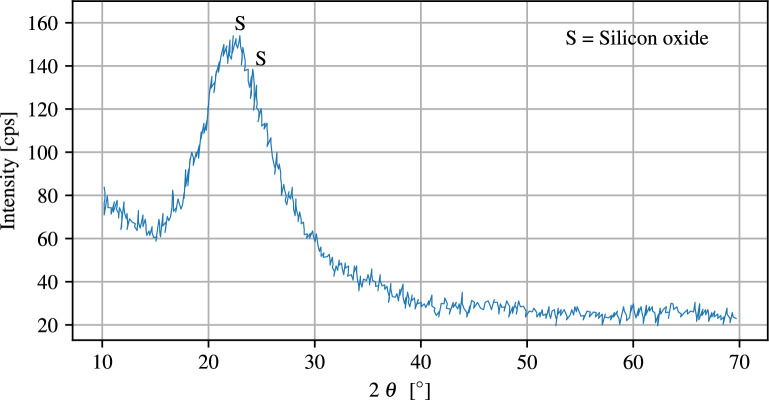
XRD of NS.

The size of NS particles with an average particle diameter of 25 nm, specific surface area of 193 $\text{m}^2/\text{g}$, and with 98% purity are purchased from Payazhic company. The composition materials and physical properties of the used NS are given in Table 3. Powder X-ray diffraction (XRD) diagram of NS nanoparticles are shown in Fig. 2.

**Table 3:** Composition materials and physical properties of NS.

$\mathrm {SiO_{2}}\ [\%]$	$\geqslant$98
$\mathrm {Al_{2}O_{3}}\ [\%]$	0.076
$\mathrm {Fe_{2}O_{3}}\ [\%]$	0.293
$\text{CaO}\ [\%]$	0.392
$\text{MgO}\ [\%]$	0.05
$\mathrm {Na_2O_3}\ [\%]$	0.328
$\mathrm {SO_3}\ [\%]$	0.185
$\mathrm {TiO_2}\ [\%]$	0.064
$\mathrm {P_2O_5}\ [\%]$	0.129
$\text{ZnO}\ [\%]$	0.021
$\text{CuO}\ [\%]$	0.020
Loss on drying $[\%]$	<5
Specific gravity $\left[ \dfrac{\text{g}}{\text{cm}^3}\right]$	2.65
Specific surface $\left[ \dfrac{\text{m}^2}{\text{g}}\right]$	193
Bulk density $\left[ \dfrac{\text{kg}}{\text{m}^3}\right]$	50
Mean particle size $[\mathrm{nm}]$	20−30
pH	5−6

**Figure 3:**
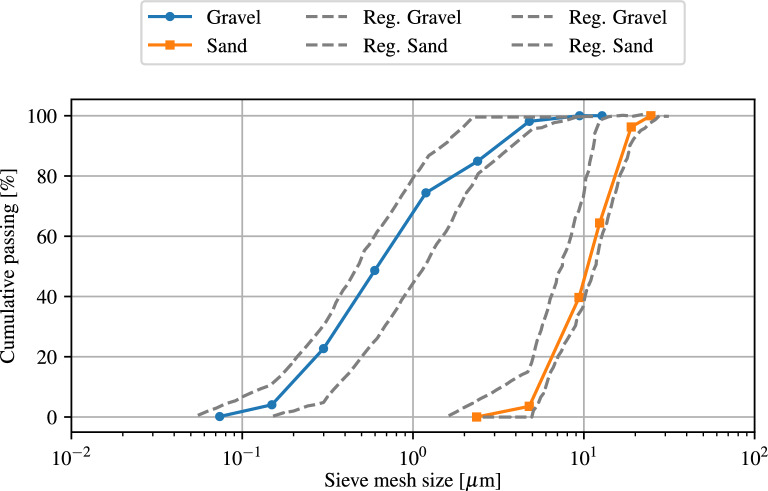
Fine and coarse aggregate grading curves with regulations limit.

Natural river sand with a fineness modulus of 2.64, specific gravity of 2.63, bulk density of 1720 $\text{kg}/\text{m}^3$, water absorption of 1.3% and a maximum size of 4.74 $\mathrm{mm}$ is used as fine aggregate. The course aggregate is crushed limestone with a maximum nominal size of 16 mm, specific gravity of 2.56, fineness modulus of 6.86, bulk density of 1580 $\text{kg}/\text{m}^3$ and water absorption of 0.78%. The fine and coarse aggregate gradation curve, shown in Fig. 3, is completely consistent with the criteria of ASTM C33^54^.

A Type F polycarboxylate acid-based superplasticizer conforming with ASTM C494^55^ is introduced in all the concrete mixtures as a weight percentage of the total cementitious materials. The superplasticizer was used to help disperse nanoparticles throughout the concrete mixture, increase compaction and improve the concrete workability. The characteristics of the superplasticizing admixture materials are given in Table 4.

**Table 4:** Composition materials and physical properties of superplasticizer.

Property	Value
Appearance	Light Brown
Freezing temperature $[^\circ \mathrm C]$	$\simeq -2$
Solid content $[\%]$	42
Specific gravity $\left[ \dfrac{\text{g}}{\text{cm}^3}\right]$	1.09

#### Chemical analysis of Caspian seawater

The water used for curing conditions in this study is obtained from the Caspian sea. To determine the chemical properties of Caspian seawater, samples of each of it were analysed. The results of the chemical analysis are given in Table 5. The concentrations of cations and anions in Caspian Seawater are not very high. Sulfate and chloride with a total quantity of 1894 ppm and 3894 ppm are only two ions which are somewhat greater than the standards.

**Table 5:** Characteristics of Caspian seawater.

	pH	Total dissolved solids	${\text{Cl}}^-$	${\text{So}}^{2-}_{4}$
	$[-]$	$[{\text{ppm}}]$	$[{\text{ppm}}]$	$[{\text{ppm}}]$
Caspian seawater	8.54	9190	3987	1894
Drinking water	7.5	572	105	159
ASTM C^56^	5–8.5	2000	1000	1000

### Concrete mix design and speciemen preparation

Three series of concrete specimens, namely Series PC and NSC Series are cast and then subjected to the tests. PC denotes plain concrete and is regarded as control concrete. The water- to- binder (the sum of cement and nano-particles) ratio (W/C) used for all mixtures is 0.5. NSC1, NSC2, NSC3, NSC4, NSC5, NSC6, NSC7, and NSC8 denote the concrete containing 1 wt %, 2 wt %, 3 wt %, and 4 wt % , 5 wt %, 6 wt %, 7 wt % and 8 wt % nanosilica, by the weight of cement, respectively. The mix proportions for one cubic meter of concrete are presented in Table 6. In order to produce the concrete including nano-particles, superplasticizer is combined with water in a mixer for 5 min before adding the nano-particles and stirring at a high speed for another 5 min. Following 2 min of low-speed mixing in a concrete centrifugal blender to establish suitable workability, the combination of water, superplasticizer, and nano-particles is gently added and swirled at a low speed for an additional 2 min to ensure proper workability. The superplasticizer is dissolved in water to make plain concrete. Water and superplasticizer are placed into a concrete centrifugal blender and swirled for several minutes to obtain optimum workability before adding the coarse aggregate, sand, and cement.

**Table 6:** Mix proportions of specimens.

Mix No.	Mixture ID	W/C	Water	Cement	NS	Fine Agg.	Coarse Agg.
		$[-]$	$\left[ \dfrac{\text{kg}}{\text{m}^3}\right]$	$\left[ \dfrac{\text{kg}}{\text{m}^3}\right]$	$\left[ \dfrac{\text{kg}}{\text{m}^3}\right]$	$\left[ \dfrac{\text{kg}}{\text{m}^3}\right]$	$\left[ \dfrac{\text{kg}}{\text{m}^3}\right]$
1	PC	0.5	175	350	–	825	1000
2	NSC1	0.5	175	346.5	3.5	825	1000
3	NSC2	0.5	175	343	7	825	1000
4	NSC3	0.5	175	339.5	10.5	825	1000
5	NSC4	0.5	175	336	14	825	1000
6	NSC5	0.5	175	332.5	17.5	825	1000
7	NSC6	0.5	175	329	21	825	1000
8	NSC7	0.5	175	325.5	24.5	825	1000
9	NSC8	0.5	175	322	28	825	1000

### Test procedures

#### Freezing and thawing

The freezing and thawing process on the PC and NSC series specimens is carried in accordance with ASTM standard C666/C666M^57^ by putting samples in a simple freezing and thawing chamber capable of attaining and maintaining the required temperature. The freezing and thawing process takes 5 hours to complete one cycle. Upon completion of one freezing and thawing cycle, the temperature changes from 4°C to -18°C and then back to 4°C. The samples was 150 mm$\times$ 150 mm$\times$ 150 mm.

#### Compressive strength

In order to conduct compressive strength tests according to ASTM C39^58^, a 200-ton hydraulic concrete pressure testing machine was employed. The specimens were filled up at 7, 14, 28, and 90 days at the rates of 100 $\text{kg}/\text{s}$ with the force-controlled load application for the compressive strength test. The size of the samples was 150 mm$\times$ 150 mm$\times$ 150 mm.

#### Water permeability and chloride ion penetration

The automatic concrete water penetration system was used to assess the water penetration depth into hardened concrete in three cells according to Ref.^59^. After making three specimens (150 mm$\times$ 150 mm$\times$ 150 mm), they were covered with a polyethylene sheet and wet burlap for 24 h prior to demoulding and curing in a water tank for 28 days. The specimens were taken out from curing tank before test and were placed in the air at 20$^{\circ }$C for 72 h. Afterwards, specimens were put in the penetration cell at 0.5 MPa water pressure for 72 h. The water penetration depth was estimated by crushing the specimens in a compression test machine using the indirect tensile method. The rapid chloride penetrability of the concrete mixtures was specified by the use of a concrete disk (a thickness of 50 mm and diameter of 100 mm) cut from a cylindrical sample with a diameter of 100 mm at curing ages of 28 days in accordance with ASTM C1202-12^60^. Total charge passed in coulombs during a 6 h test represents the concrete resistance to chloride ion penetration.

### Phase-field models for crack propagation

#### Governing equations

The numerical description of fracture processes is a challenging task, and part of ongoing research. Focusing on brittle materials^44^ proposed and energetic approach, minimising the total energy of a (fractured) system, which consists of the elastic energy of the undamaged solid and the fracture energy, based on Griffith theory^61^. To overcome difficulties within the minimisation due to the discontinuous displacement field^62^, introduced an order parameter, the phase-field, leading to a volumetric representation of the crack surface alongside with a spatially continuous displacement field.

The set of governing equations follows from minimisation of a total energy potential $\Pi _\text{tot}$^63^:

1 $$\begin{aligned} \underset{{\textbf{u}},\,\text{d}}{\mathrm {arg\,min}}\;\Pi _\text{tot} \end{aligned}$$

Considering an spacial domain $\Omega$ with fixed displacements on $\partial \Omega _D$ and traction forces on $\partial \Omega _N$ this energy can be expressed as

2 $$\begin{aligned} \Pi _\text{tot} = \int _{\Omega _0} \rho _0\psi \;\text{dV} - \int _\mathrm {\partial \Omega _N} {\textbf{t}}\cdot {\textbf{u}}\;\text{dA} + \int _{\Omega _0} \frac{\mathrm {g_c}}{2}\left[ \frac{\text{d}^2}{\mathrm {l_c}}+\mathrm {l_c}\,\nabla _{{\textbf{X}}}\left( \text{d}\right) \cdot \nabla _{{\textbf{X}}}\left( \text{d}\right) \right] \;\text{dV}. \end{aligned}$$

The internal energy energy of the bulk is expressed within a spectral decomposition^45^. As energy is dissipated during crack evolution, the internal energy of the undamaged solid is degraded with evolving fracture. Applying the so called degradation function $g(\text{d})$ only onto the tensile part of the Helmholtz-energy $\rho _0\,\psi ^+$, limits crack formation to tension:

3 $$\begin{aligned} \rho _0\,\psi = \underbrace{(1 - \text{d})^2}_{g(\text{d})}\;\rho _0\,\psi ^+ + \rho _0\,\psi ^- \quad \text {with}\quad \rho _0\,\psi ^\pm = \tfrac{1}{2}\lambda \,\left<\text{tr}\left( \pmb {\varepsilon }\right) \right>_\pm ^2 + 2\mu \,\text{tr}\left( \pmb {\varepsilon }\,\pmb {\varepsilon }\right) \end{aligned}$$

Compleering Eq. (3) the definitions of the so called makula-bracket

4 $$\begin{aligned} \left<\cdot \right>_+=\text{max}(0, \cdot ) \quad \text {resp.}\quad \left<\cdot \right>_-=\text{min}(0, \cdot ) \end{aligned}$$

as well as the tensile- as well as compressive strains are required. With $\lambda ^i _\varepsilon$ resp. ${\textbf{n}}^i _\varepsilon$ denoting the eigenvalues resp. the eigenvectors of $\pmb {\varepsilon }$ the decomposed strains follows as

5 $$\begin{aligned} \pmb {\varepsilon }^\pm = \sum _{i=0}^3 \left<\lambda ^i _\varepsilon \right>_\pm \;{\textbf{n}}^i _\varepsilon \otimes {\textbf{n}}^i _\varepsilon . \end{aligned}$$

Evaluating the minimisation problem (1) based on Eq. (2) – the stress is defined as the derivative of the Helmholtz energy with respect to strain $\pmb {\sigma }^\pm =\partial \rho _0\,\psi ^\pm /\partial \pmb {\varepsilon }^\pm$ – leads to

6 $$\begin{aligned} \begin{aligned} \nabla _{{\textbf{X}}}\cdot \left[ g(\text{d})\,\pmb {\sigma }^+ + \pmb {\sigma }^-\right]&= {\textbf{0}}\\ \mathrm {g_c}\,\left[ \frac{\text{d}}{\mathrm {l_c}}-\mathrm {l_c}\,\Delta _{{\textbf{X}}}\cdot \left[ \text{d}\right] \right] -2(\text{d}- 1)\,\rho _0\,\psi ^+&=0 \end{aligned} \end{aligned}$$

To ensure the irreversibility of crack-growth, the crack-driving-force $2\left( \text{d} - 1\right) \rho _0\psi ^+({\textbf{x}},\,\text{t})$ is replaced by a so called history term^46^

7 $$\begin{aligned} 2\left( \text{d} - 1\right) \,{\mathscr {H}}({\textbf{x}},\,\text{t}) \quad \text {with}\quad {\mathscr {H}}({\textbf{x}},\,\text{t})=\underset{\tau \in [0,\,\text{t}]}{\text{max}}\hspace{0.2cm}\rho _0\psi ^+({\textbf{x}},\,\tau ). \end{aligned}$$

#### Discretisation and solution strategy

For the solution of Eqs. (6) a standard Galerkin finite-element-method is used. Let $\Omega _0$ be the reference placement of a domain with non-overlapping Dirichlet- and Neumann boundaries $\partial \Omega _{0,D}$ resp. $\partial \Omega _{0,N}$. Thereon the weak solutions $({\textbf{u}},\;\text{d})\in \text{V}_{\textbf{u}}\times \text{V}_\text{d}$ with

8 $$\begin{aligned} \begin{aligned} \text{V}_{\textbf{u}}\left( \Omega _0\right)&=\left\{ u\in \text{H}^1\left( \Omega _0\right) :\; {\textbf{u}}={\textbf{u}}_D\;\text {on}\;\partial \Omega _{0,D}\right\} \quad \text {resp.}\\ \quad \text{V}_\text{d}\left( \Omega _0\right)&=\left\{ \text{d}\in \text{H}^1\left( \Omega _0\right) :\; \text{d}\in [0,1]\right\} \end{aligned} \end{aligned}$$

have to fulfill

9a $$\begin{aligned}&\int _{\Omega _0} \left[ g(\text{d})\;{\varvec{\sigma }}^+ + {\varvec{\sigma }}^- \right] \cdot \pmb {\varepsilon }({\textbf{v}}_\text{u})\;\text{dV}= \int _{\partial \Omega _0} {\textbf{t}}\cdot {\textbf{v}}_\text{u}\;\text{dA} \end{aligned}$$

9b $$\begin{aligned}&\int _{\Omega _0} \left[ \frac{\mathrm {g_c}}{\mathrm {l_c}} \text{d}- 2(\text{d}- 1){\mathscr {H}}({\textbf{x}},\,\text{t})\right] \,\text{v}_\text{d}+ \mathrm {g_c}\mathrm {l_c}\,\nabla _{{\textbf{X}}}\left( \text{d}\right) \cdot \nabla _{{\textbf{X}}}\left( \text{v}_\text{d}\right) \;\text{dV}=0 \end{aligned}$$

for all test-functions $({\textbf{v}}_{\textbf{u}},\;\text{v}_\text{d})\in \text{V}_{\textbf{u}}\times \text{V}_\text{d}$. We assumed homogeneous Neumann boundary condition on $\partial \Omega _0$ for the phase-field within Eq. (9b).

This coupled system is the solved, using a staggered scheme^62^ (see algorithm 1), as the monolithic approach suffers with respect to stability when rapid changes of the phase-field are present. Therein phase-field and displacement are calculated sequentially, until – after the updated displacement is calculated – the residual of the phase-field reaches a prescribed tolerance. A widely adopted strategy^46^ can be seen as a special case of this algorithm, limiting the number of staggered iterations $n_{maxStag}$ to one. This limitation mainly reduces the crack evaluation speed after the phase-field reaches one, while significantly reduces the computational costs.

**Algorithm 1:**
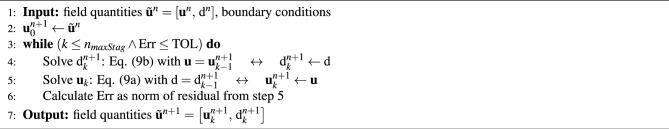
Staggered algorithm based on alternating minimization.

A validation of the method is provided in the Supplementary Material.

## Results and discussion

### Mass loss and length reduction

**Figure 4:**
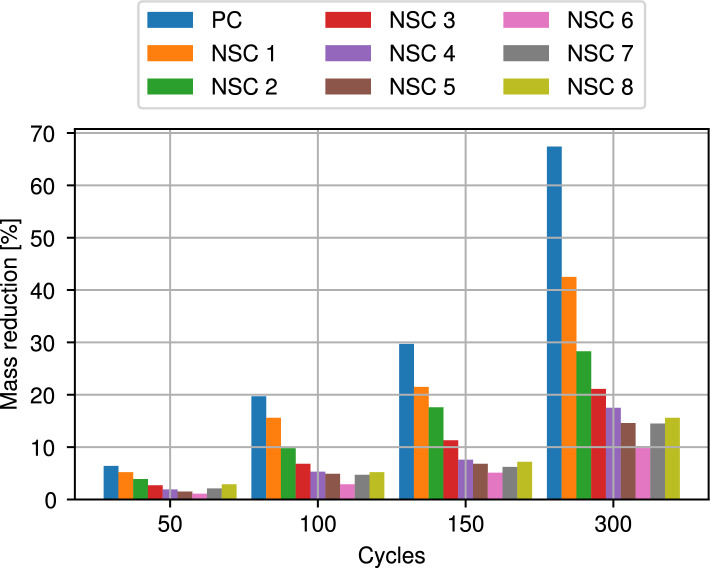
Mass loss in specimens after freeze and thaw cycles with respect to initial specimen.

In order to determine the frost resistance of the concretes, mass loss and changes in specimen length are measured after 50, 100, 150, and 300 cycles of freezing and thawing. The results for the mass loss are summarized in Table 7 and visualized in Fig. 4.

**Table 7:** Mass loss in specimens after freeze and thaw cycles with respect to initial specimen.

Mix No.	Mixture ID	50 cycles		100 cycles		150 cycles		300 cycles	
		$[\%]$	$[\mathrm g]$	$[\%]$	$[\mathrm g]$	$[\%]$	$[\mathrm g]$	$[\%]$	$[\mathrm g]$
1	PC	6.4	534.3	19.7	1644.9	29.7	2479.8	67.4	5627.7
2	NSC1	5.2	435.2	15.6	1305.7	21.5	1799.5	42.5	3557.2
3	NSC2	3.9	327.1	9.8	821.9	17.6	1476.1	28.3	2373.4
4	NSC3	2.7	226.8	6.8	571.2	11.3	949.2	21.1	1772.4
5	NSC4	1.9	159.9	5.3	446.1	7.6	639.7	17.5	1473.1
6	NSC5	1.5	126.4	4.9	413.1	6.8	573.2	14.6	1230.8
7	NSC6	1.1	93.1	2.9	245.4	5.1	431.6	10.2	863.3
8	NSC7	2.1	178.2	4.7	398.9	6.2	526.2	14.5	1230.7
9	NSC8	2.9	246.6	5.2	442.2	7.2	6.12.3	15.6	1326.7

**Figure 5:**
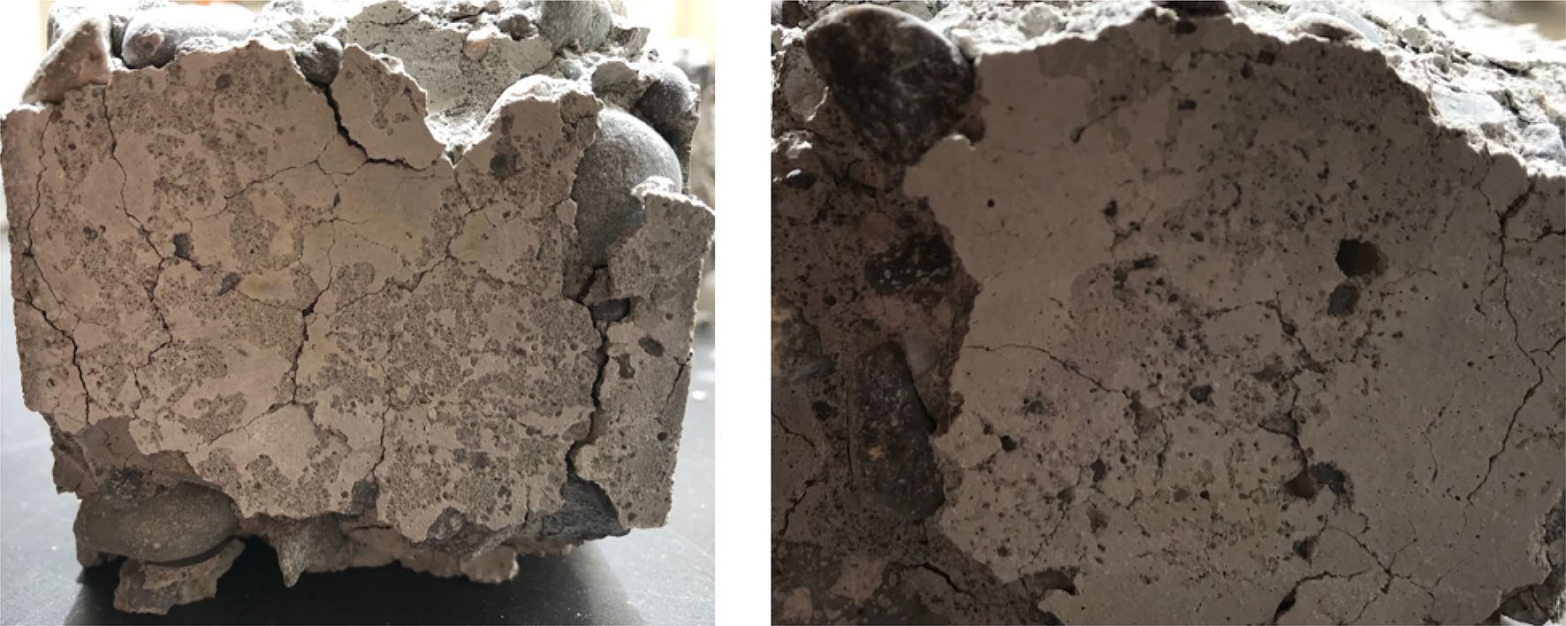
Pictures of used concrete after freezing and thawing cycle with resulting mass loss.

Whereas the original specimens have a rectangular cross-section, after experiments clear spalling with a significant loss of mass is evident. This mass loss from concrete under freezing and thawing cycles compared to the initial sample occurs due to damages in the edges of each specimen. Our measurements show, that mass loss reduces by increasing the amount of NS up to 6%, but increased thereafter. This behavior becomes also apparent by comparing the length reduction of the specimen after freezing and thawing cycles. Table 7 and Fig. 4 illustrate that the length reduction decreases with a higher mount of NS and results in a maximum at 6% NS. This coincides with the previous results on weight loss very closely. This behavior does not change with the number of cycles.

The use of NS can contribute to reduce the impact of freezing and thawing cycles on concrete structures by changing the pores and decreasing the porosity of the concrete so that less water penetrates the concrete. Since the concrete with additional NS has a more effective pore structure than standard mixes, the resulting concrete is more durable and the capillary pore size is strengthened at the conclusion of thaw cycles, which causes a smaller percentage weight loss for the NS concrete. The loss of mass of a specific concrete sample can be seen very clearly in Fig. 5, in which large parts have burst out of the previously straight block, creating large holes and uneven edges. Cheng et al. reported that the rate of mass loss in nano-$\mathrm{SiO_2}$ concrete was lower than in concrete without nano-$\mathrm{SiO_2}$ for up to 300 freeze-thaw cycles. Additionally, the rate of loss decreased as the number of cycles increased in both non-chloride salt and chloride salt environments^64^.

### Compressive strength

**Figure 6:**
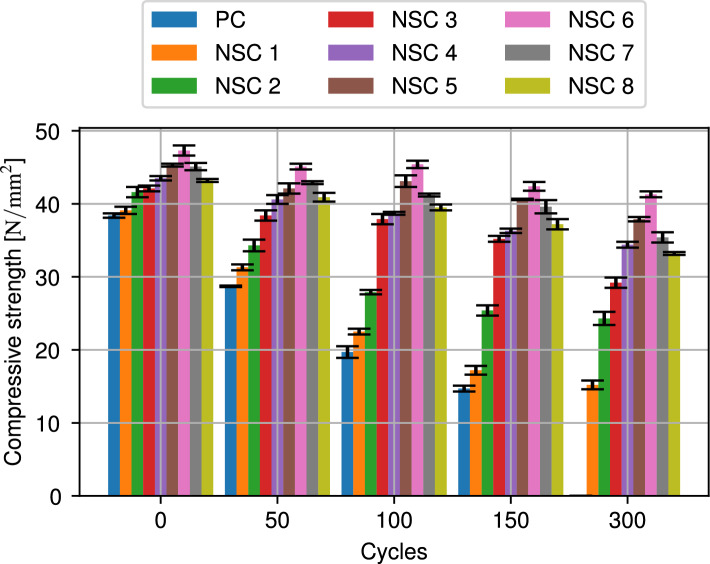
Compressive strength of specimens after freeze and thaw cycles.

Based on different studies^65–68^, it has been found that even a small amount of nano-$\mathrm{SiO_2}$ in concrete during its early stages can greatly improve its compressive strength. An additional aim of this study was the determination of the effect of freezing and thawing cycles on the compressive strength. The corresponding values for concretes with different amounts of additive and different cycles are shown in Table 8 and illustrated in Fig. 6.

**Table 8:** Compressive strength of specimens after freeze and thaw cycles.

Mix No.	Mixture ID	Initial	50 cycles	100 cycles	150 cycles	300 cycles
		$\left[ \dfrac{\text{N}}{\text{mm}^2}\right]$	$\left[ \dfrac{\text{N}}{\text{mm}^2}\right]$	$\left[ \dfrac{\text{N}}{\text{mm}^2}\right]$	$\left[ \dfrac{\text{N}}{\text{mm}^2}\right]$	$\left[ \dfrac{\text{N}}{\text{mm}^2}\right]$
1	PC	38.4 ± 0.3	28.7 ± 0.1	19.7 ± 0.8	14.7 ± 0.4	0
2	NSC1	39.1 ± 0.5	31.3 ± 0.4	22.5 ± 0.4	17.2 ± 0.6	15.2 ± 0.6
3	NSC2	41.6 ± 0.7	34.4 ± 0.8	27.9 ± 0.3	25.4 ± 0.7	24.3 ± 0.9
4	NSC3	42.1 ± 0.4	38.4 ± 0.7	37.9 ± 0.7	35.2 ± 0.4	29.2 ± 0.7
5	NSC4	43.5 ± 0.3	40.6 ± 0.6	38.7 ± 0.2	36.3 ± 0.3	34.4 ± 0.4
6	NSC5	45.3 ± 0.2	42.1 ± 0.7	43.1 ± 0.8	40.6 ± 0.1	37.9 ± 0.3
7	NSC6	47.3 ± 0.7	45.1 ± 0.4	45.4 ± 0.5	42.4 ± 0.6	41.3 ± 0.4
8	NSC7	45.1 ± 0.5	42.9 ± 0.2	41.2 ± 0.2	39.6 ± 0.9	35.4 ± 0.7
9	NSC8	43.2 ± 0.2	40.9 ± 0.6	39.5 ± 0.4	37.2 ± 0.7	33.2 ± 0.2

Compressive strength test was done by loading specimens after 90 days curing and subjecting to uniaxial load until failure. Compressive strength of concrete containing NS increases as NS content approaches the threshold. A higher amount of NS over the threshold value reduces compressive strength^36^. The primary factor behind enhancing the compressive strength of concrete containing NS is the pozzolanic reaction caused by a reaction between NS and calcium hydroxide. This reaction facilitates the creation of hydrated calcium silicate, which contributes to the overall strength of the concrete. The early improvement in compressive strength of concrete with NS is more pronounced, attributable to the heightened pozzolanic activity of NS particles^69,70^. Nevertheless, the postponement of the curing period resulted in a steady decline in the presence of NS particles in the pozzolanic reaction, thus diminishing the subsequent enhancement of concrete’s compressive strength^71^. Adding NS needs to be strictly controlled regarding optimum admixture, as excessive NS agglomeration occurs, gradually decreasing compressive strength. Adding NS needs to be strictly controlled regarding optimum admixture, as excessive NS agglomeration occurs, gradually decreasing compressive strength. Furthermore, adding NS over optimum admixture leads to a reduction of compressive strength as the nano-doping increases^72^. It can be seen that NS has also a favorable effect on the compressive strength of concrete, during freezing-thawing cycle. Compared to the control samples, the increase in NS increases the compressive strength during freezing-thawing cycles. Moreover, concrete could have up to 6% more NS added to it to reduce the amount of compressive strength loss compared to before the freezing-thawing cycle. For example, a compressice strength reduction of 48.93% was measures after 100 cycles in the control samples, whereas this reduction reduces to 32.93% with 2% NS, 11.03% with 4% NS and finally to the lowest loss of 4.02% with 6% NS. However, increasing the amount of NS by more than 6% indicates the opposite effect on the compressive strength of concrete during freeze and thaw cycles. This can be explained by crack nucleation speeding up the hydration process due to its large specific surface area. Furthermore, due to its fineness and high silica concentration, NS is an excellent reactive pozzolanic material for concrete. The NS particles agglomerated, which prolonged the amount of time it required for their reaction with excess calcium hydroxide (CH) to generate CSH gel. They also served as filler materials, which decreased porosity and improved early age strength^73^. Belkowitz et al.^74^ found that cement composite containing larger nano-$\mathrm{SiO}_2$ particles showed a significant increase in compressive strength and elastic modulus, exceeding 20% compared to the reference mixtures. Zhao et al.^40^ performed an experiment to examine the correlations between the nano-$\mathrm{SiO}_2$ content of nano-$\mathrm{SiO}_2$ concrete, air content, water-cement ratio, and frost resistance property. Their findings demonstrate that incorporating nano-$\mathrm{SiO}_2$ into concrete enhances its resistance to freezing-thawing cycles and compression strength. This improvement is observed as the number of impermeable voids in the hardened cement paste increases.

### Water permeability and chloride ion penetration

**Figure 7:**
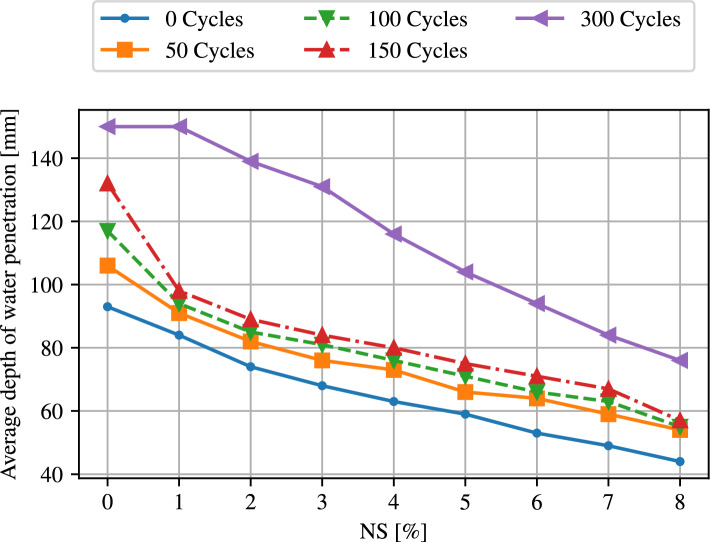
Average depth of water penetration for 0, 50, 100, 150 and 300 freezing and thawing cycles for different percentages of NS.

The water penetration of concrete with various amounts of NS was studied for different freezing-thawing cycles to investigate the material changes resulting from the procedure. Figure 7 depicts the average depth of water penetration for normal concrete as well as concrete with different amounts of NS for different numbers of freezing-thawing cycles. First, it can be observed that the water depth decreases significantly with an increasing amount of additives. The highest water depth is reached with normal concrete. In addition, it becomes evident that the depth also increases with an increase in the number of cycles, so that the values after 300 cycles are significantly higher than within the initial sample.

**Figure 8:**
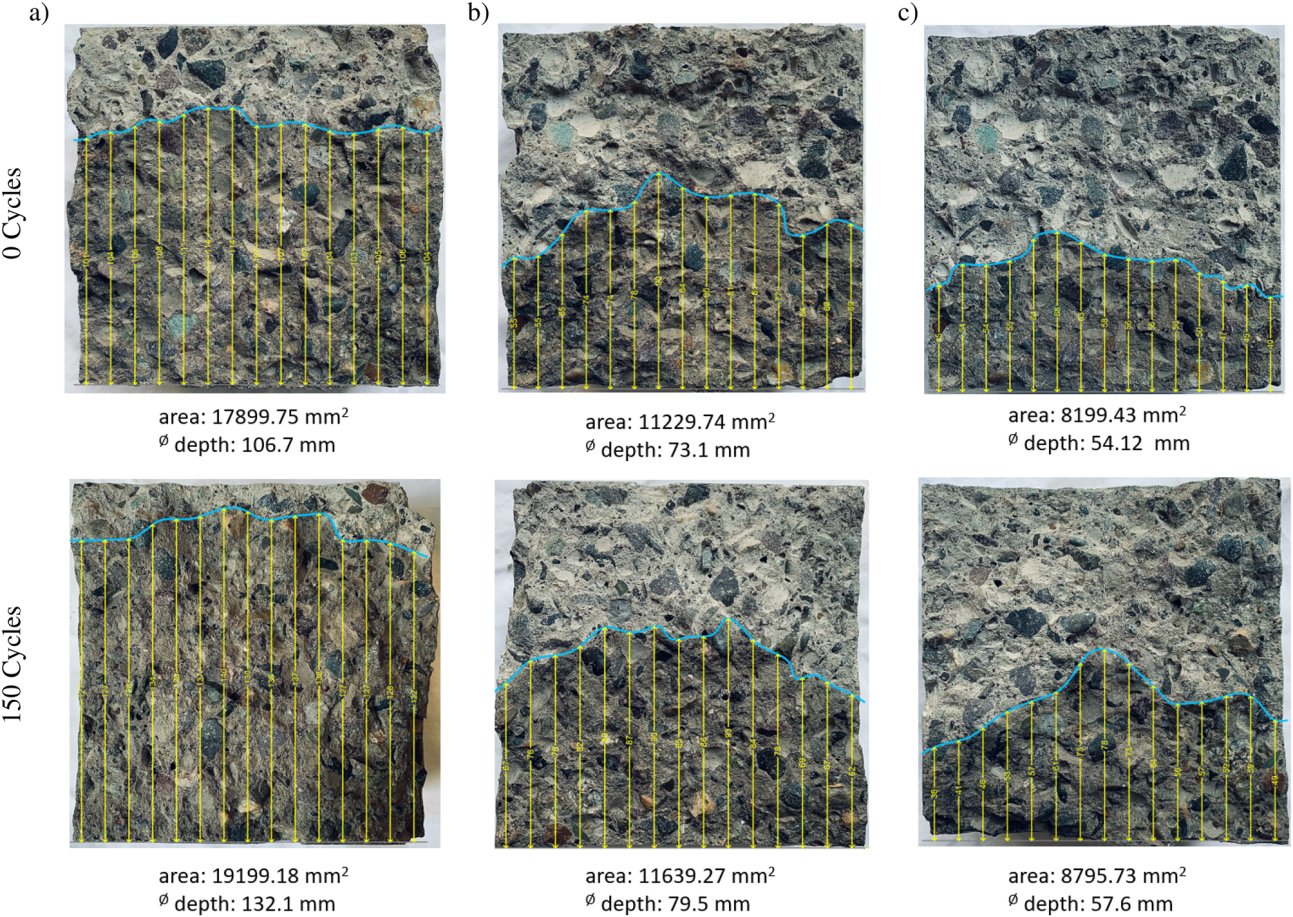
Water penetration for concrete with (**a**) normal concrete, (**b**) 4% NS and (**c**) 8% NS for 0 cycles (upper row) and 150 cycles (lower row) with indication of the area of water penetration and the average water penetration depth for each specimen.

Figure 8 displays the formation of the water front within a concrete cross-section at different cycles. In addition, the corresponding height of this front from the bottom of the cross-section is shown. It underlines the above findings. The height of the water penetration is increase after freezing and thawing cycle compared to the initial state for each specimen. Also it is apparent, that the height changes with different percentages of NS, where a high percentage of NS causes a lower water penetration.

The reduction of penetration depth is a consequence of the already discussed reduction of the permeability. This structure enables the absorption of the free water, which occurs in the mixing of the concrete. In addition, compared to PC, the interfacial transition zone thickness around the coarser size aggregate is less in concrete with NS. The reduction in water penetration caused by the inclusion of NS as compared to the reference concrete mix suggests that the capillary pores’ ability for interaction with adjacent ones was severely weakened by the pores’ increased filling of hydration products brought on by NS’s high pozzolanic reactivity. Elkady et al.^73^, Erdam et al.^75^, and Rezania et al.^76^ observed that the permeability reduction exhibited a declining pattern as the nanoparticle content increased. This was attributed to the challenges in dispersing the nanoparticles in concrete at higher dosages. The utilization of nano-$\mathrm{SiO}_2$ in alkali-activated slag concrete resulted in an unfavorable increase in water penetration depths. Conversely, the incorporation of nano-$\mathrm{SiO}_2$ decreased the permeability^77^.

**Figure 9:**
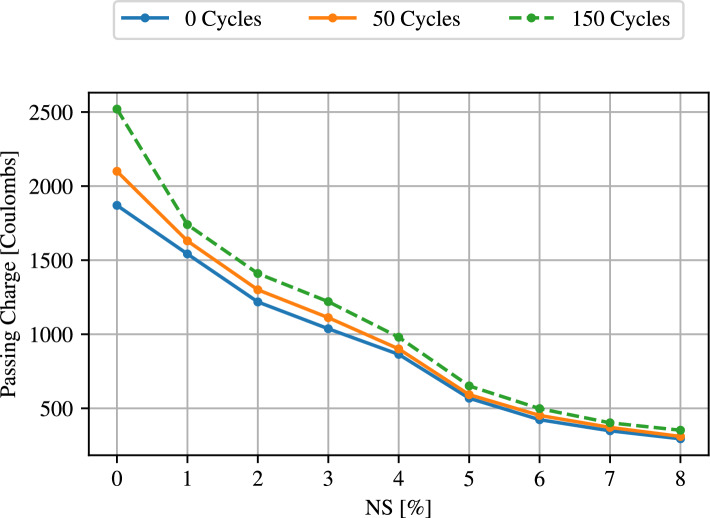
Rapid chloride penetrability for 0, 50, and 150 freezing and thawing cycles for different percentages of NS.

As another important value for concretes, the chloride ion penetration at different cycles was investigated. Figure 9 illustrates the passing charge for normal concrete and different additions of NS after again 0, 50 and 150 freezing and thawing cycles.

The evaluation of the rapid chloride penetrability also shows a decrease of the passing charge with increasing proportion of NS in the concrete. The highest value is also reached with normal concrete, while the passing charge drops sharply up to a value of 6% NS and from there reaches a flatter descent. Furthermore, this evaluation also reveals an increase in passing charge with a higher number of freezing and thawing cycles. However, the differences between the cycles become smaller as the amount of added NS increases. The high NS dosage (3–8%) may cause concrete’s fast chloride penetrability to fall from a low grade to a very low rating when replacement levels drop. The volume of large pores and the total amount of porosity in the concrete can be decreased with an increase in NS content and fineness. As a result, deeper water penetration and chloride ion penetration are reduced by an increase in the percentage of NS. The lowering in the critical threshold diameter of pores and the finer microstructure brought on by the addition of NS can both contribute to the reduction of charge transferred inside the concrete^65^. With an increase in NS, the threshold and critical diameters of the pores as well as the big capillary porosity decreased^65^. Isfahani et al.^78^ and Jala st al.^69^ revealed that using nano-$\mathrm{SiO}_2$ can reduce chloride ion penetration significantly. The reduced conductivity in concrete with a small dose of nano-$\mathrm{SiO}_2$ resulted in lower chloride ion penetration in the cementitious matrix^79^. The resistance to penetration of chloride ions in the nano-$\mathrm{SiO}_2$ concrete was relatively high compared to nano-$\mathrm{SiO}_2$ and reference concretes^80^.

### Simulation of crack propagation

Besides the experimental results, the resistance of concrete against cracks before and after freezing and thawing cycles will be further investigated by numerical simulations. We therefore apply the phase-field method, presented in section "Phase-field models for crack propagation". First, an appropriate boundary value problem is determined, which is tailored to the real conditions of a representative sample. Subsequently, the required fineness of the mesh is obtained by means of a convergence study. This setup is then used to determine the behaviour of this representative sample under compression. As shown within the previous sections, material parameters of concrete are highly affected by the number of freezing-thawing cycles. The effect of these changes onto the crack resistance is discussed.

#### Boundary value problem

**Figure 10:**
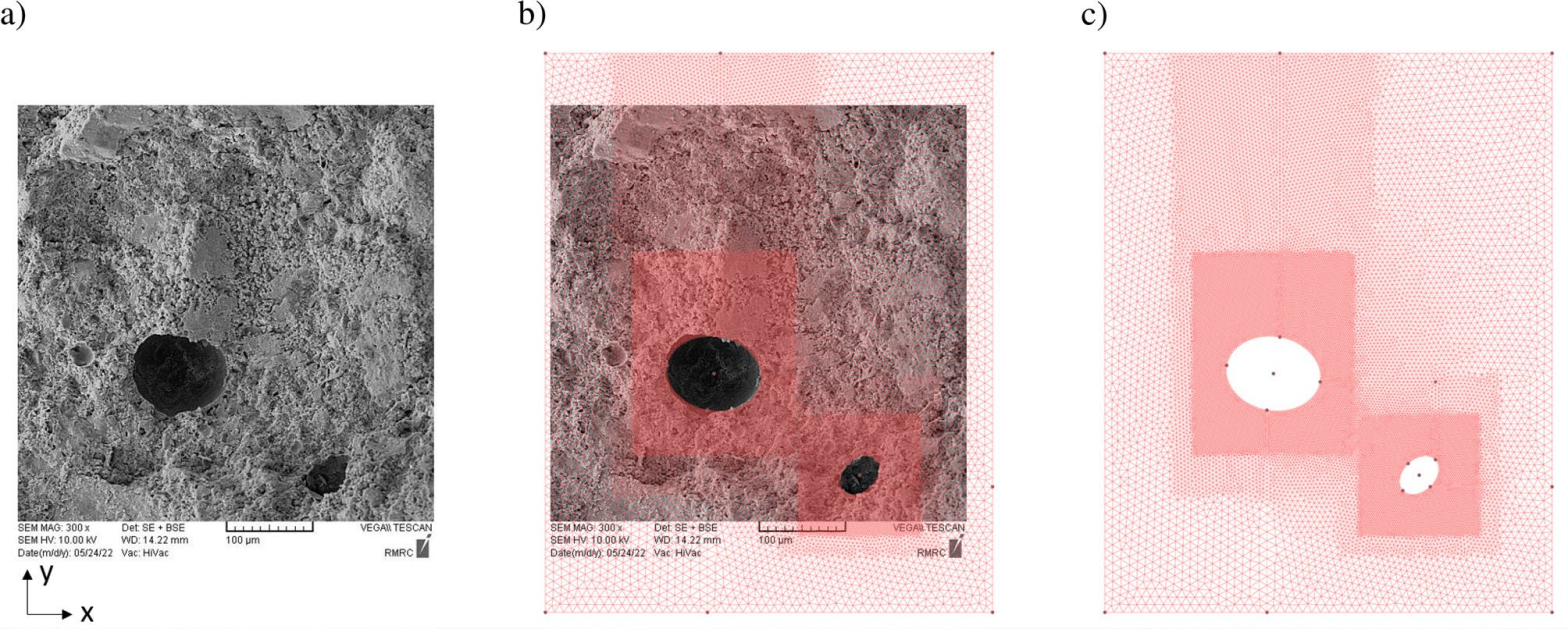
Deduction of the geometry and meshing based on a microscope section. (**a**) Microscope image of concrete with inclusion. The image was generated with 300x SEM MAG, 10.00 kV SEM HV and a working distance of 14.22 mm. (**b**) Overlay of meshing with microscopic image. (**c**) The resulting mesh with refined areas around the inclusions.

The boundary value problem is derived from microscope images of a concrete sample. The observed inclusions are included in the FE mesh as cutouts. To avoid (numerical) boundary effects, a sufficiently large area around the inclusions is simulated. Since cracking is expected in the area of the inclusions, locally refined triangulations are used to capture this zones properly. The respective microscope image and the resulting mesh can be seen in Fig. 10.

Based on the so far described geometry, boundary conditions are chosen as follows: On the lower side of the domain vertical displacements are set to zero. Additionally the horizontal displacement is set to zero in the bottom-right corner. A further displacement condition is applied on the top of the domain. By prescription of a negative, vertical displacement the domain is compressed, leading to crack formation near the cutouts. For the simulation we chose $\mathrm g_c = 0.0027\:\mathrm{kN/mm}$^81^ and the Poisson ratio $\nu = 0.3$.

#### Spatial crack propagation

**Figure 11:**
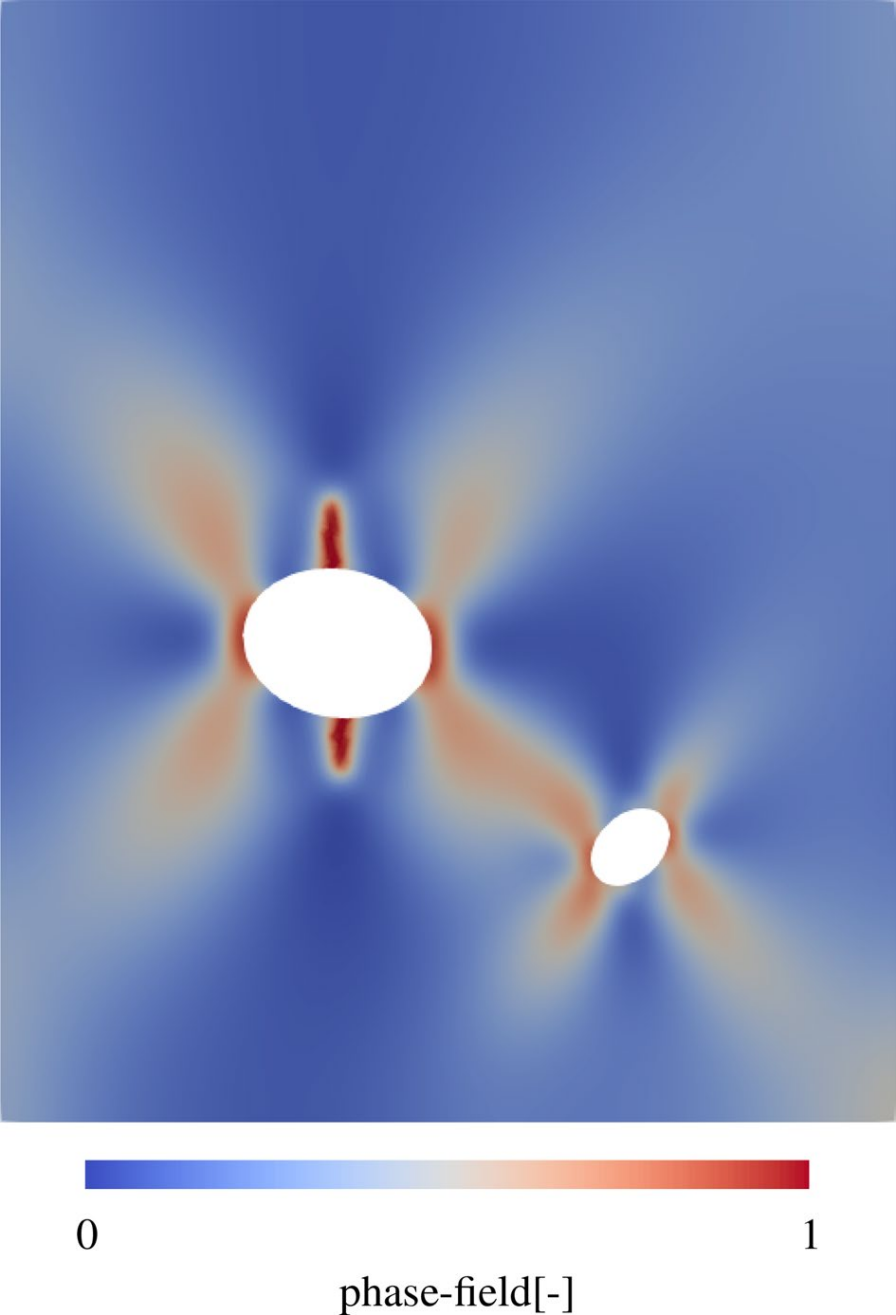
Spatial evaluation of the phase-field, representing the crack propagation in a concrete sample.

Based on the described boundary value problem, crack propagation is calculated. The spatial distribution of the phase-field – 1 indicates an entirely damaged state – is shown in Fig. 11. Under load, the concrete begins to crack at the upper ends of the large inclusion, where tensile forces are maximum. This crack propagates into vertical direction, while further cracks starts forming at the sides of the larger inclusion. It can be shown that the phase-field increases in diagonal directions from the inclusions and connects between the two inclusions.

The crack propagation is comparable to literature results^82^. This paper simulates a compression test for concrete with inclusions, but uses an anisotropic description of the concrete. Here, the spatial structure of the main vertical cracks, as well as larger areas in the 45° angle of the phase-field, is also visible. However, the simulation still shows significant blurring in the formation of the crack and the crack edges. This indicates that the so far considered mesh shows reasonable results for crack propagation, but these might still be improved with a finer mesh. For this reason, a convergence study will be performed to determine the appropriate mesh size and thus improve the accuracy of the results.

#### Convergence study

The determination of the optimal mesh fineness requires a convergence study based on subsequently refined meshes. Based on the mesh shown in Fig. 10, the characteristic mesh size for the finest region is gradually decreased.

**Figure 12:**
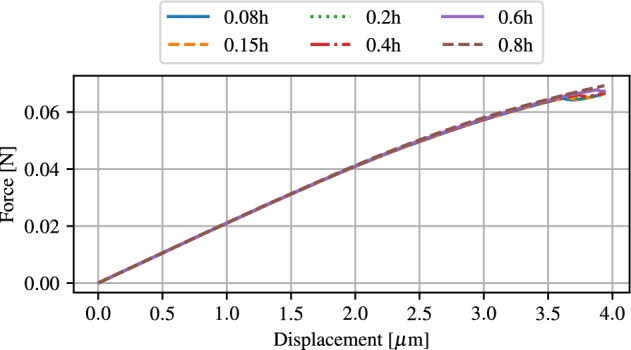
Force plotted over displacement for different characteristic lengths of the mesh.

First, the force-displacement curve is evaluated for six different refinements, as shown in Fig. 12. It is clear that the results show a sufficiently accurate similarity for all refinements and there is only a small difference in the resulting force for a given displacement. The force-displacement relationship is thus not dependent on the mesh and is therefore not a suitable criterion to determine the mesh size.

**Figure 13:**
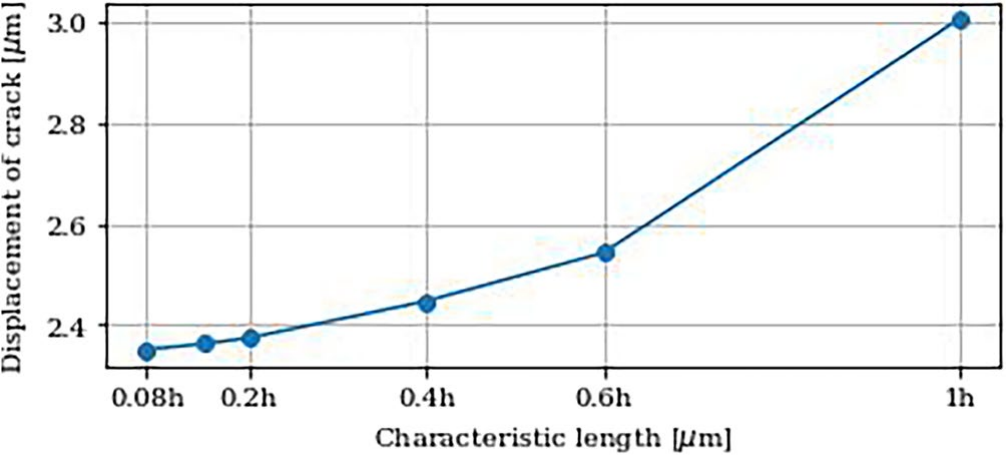
Critical displacement of crack initialisation for different characteristic lengths of the mesh.

Nevertheless, crack propagation by phase-field method is strongly mesh dependent. For this reason, the displacement at which the initial crack appears, is investigated. For each refinement, this displacement is graphically determined and plotted against the characteristic length of the elements in the refined mesh area, see Fig. 13. The evaluation of the critical displacement shows a clear convergence with increasing mesh resolution. Here, the displacement settles in an acceptable range starting at a value of about 0.2h, in which the displacement does not significantly decrease further, even with increasing mesh fineness. In addition to the critical displacement, the spacial distribution of the phase-field also play an important role in the evaluation of the crack behaviour.

**Figure 14:**
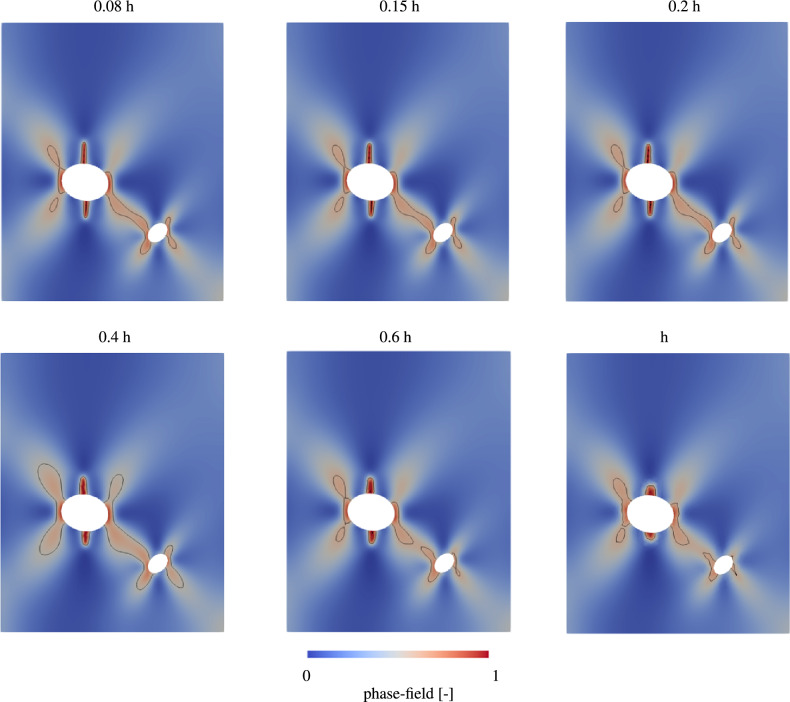
Spatial evaluation of crack propagation for different characteristic lengths of the mesh. Isolines denote a phase-field of 0.6 and 1.

For this reason, the spatial distribution of the crack and its accuracy at a given time are additionally investigated for different meshes. Figure 14 shows the crack propagation under a defined displacement of 3.0375 $\mu\mathrm{m}$ for different mesh sizes.

**Figure 15:**
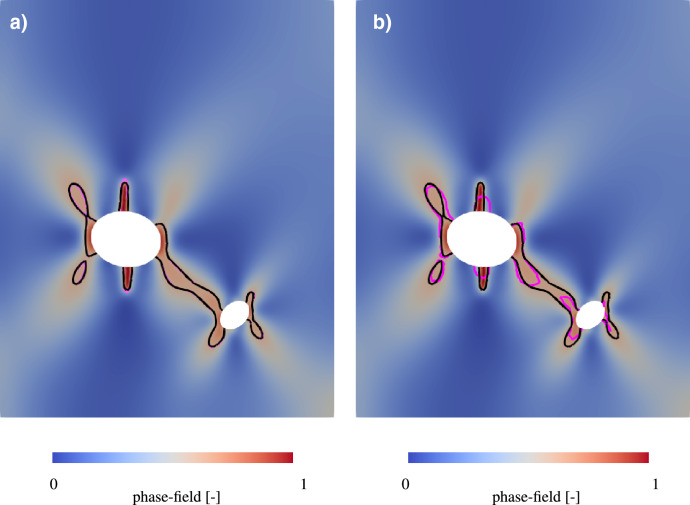
Spatial evaluation of crack propagation with isolines denoting a phase-field of 0.6 for a (**a**) a mesh size of 0.08h (magenta) and 0.2h (black) and (**b**) a mesh size of 0.2h (black) and 11.82 (magenta).

Here, the distribution of the phase-field within the examined region is shown. Since the displacement is above the critical displacement, the formation of a crack is clearly visible. For further investigation of this crack, the isolines indicates phase-field values between 0.6 and 1.0. The spatial evaluation confirms the results of the previous analysis. In the results of the upper row, the crack is illustrated with clear contours. In addition, the isolines for all three evaluations show a very similar course. However, if the results are compared with the lower row and thus with the coarser meshing, a large difference becomes apparent. First, the crack is significantly smaller despite the same displacement. Furthermore, the crack is clearly less defined and has blurrier contours. The isolines also differ greatly in both shape and size from those with the finer meshing. The difference in isolines can also be clearly seen in Fig. 15, where the spatial distribution for the mesh with a mesh size of 0.2h is shown and isolines for this mesh compared to a finer mesh on the left and a coarser mesh on the right are displayed. The isolines on the left figure show a good agreement and the differences are negligable. In contrast, the comparison with the coarse mesh shows significant differences in the results and widely varying isolines. Since these overlays show a great variance, it can be concluded, that the coarse sized mesh is not sufficient to deliver accurate results. It is therefore evident that the meshsizes 1h, 0.6h and 0.4h do not accurately reflect the crack propagation and are therefore not suitable for our calculation. As an optimal compromise between the accuracy of the expected results and the required computing time, a characteristic length of 0.2h in the finest area of the mesh around the inclusions is therefore selected.

### Influence of $\mathrm g_c$ on crack length

**Figure 16:**
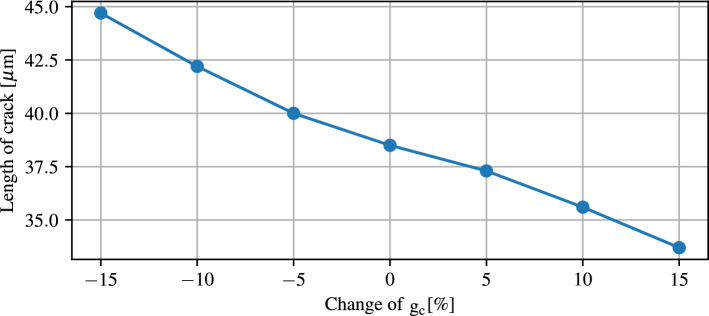
Evaluation of the influence of changes in the parameter $\mathrm g_c$.

The crack propagation simulation is based on Griffith theory, where it is not possible to measure the $\mathrm g_c$ parameter. since various $\mathrm g_c$ parameters are used in literature, the influence of the parameter $\mathrm g_c$ on the crack will be investigated numerically. Figure 16 displays the different crack lengths for a given displacement with variation of the parameter $\mathrm g_c$.

The curve clearly indicates that the crack length for the same displacement decreases significantly with an increase in the parameter. This development shows an almost linear behaviour over the range with a variation of -15% to 15% of the current value.

### Influence of compressive strength on crack length

An increasing number of freezing and thawing cycles in concrete leads to a reduction in compressive strength, as described in section "Compressive strength". Influences of this reduction on the fracture resistance is investigated numerically, using phase-field simulation. For this purpose, the cracking behaviour of nine mixtures of concrete is simulated within their initial state and after 150 cycles, using the respective compressive strengths from Table 8.

For all simulations within this section, the Dirichlet condition on the top-surface is replaced by an compressive force. First, the influence of a changed compressive strength on the force required for crack initiation is investigated. For this purpose, the compressive strength is varied in the range from 20 N/mm$^2$ to 55 N/mm$^2$ and the corresponding force is determined, by integration of the traction vector over the top-surface. It should be mentioned that the compressive strength for load-bearing concrete is above 25 N/mm$^2$, but a larger range is initially selected in the numerical study in order to represent all the effects of the model.

**Figure 17:**
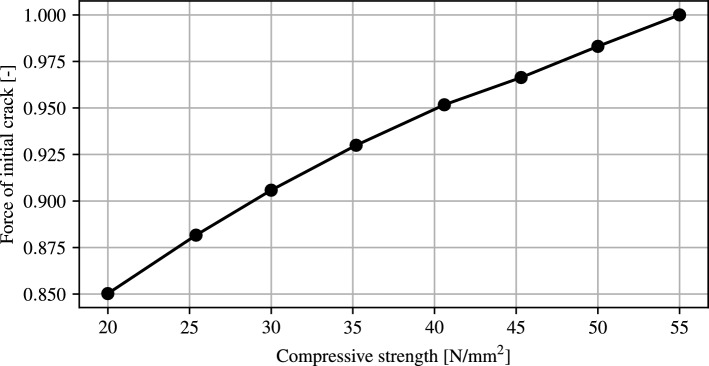
Evaluation of the force required for an initial crack for different compressive strengths.

In Fig. 17 the crack initialization force normalized to the maximal force is plotted against the compressive strength. It is evident that an increase in compressive strength leads to an increase in the crack initialisation force. This can be explained by the higher stiffness, and resulting therefrom a lower strain energy. As this is the crack-driving-force, higher forces have the be applied before fracture occurs. The increase in the crack-initialisation-force is slightly nonlinear due to the quadratic dependency of the strain energy onto the linearized Green-Lagrange strain.

**Figure 18:**
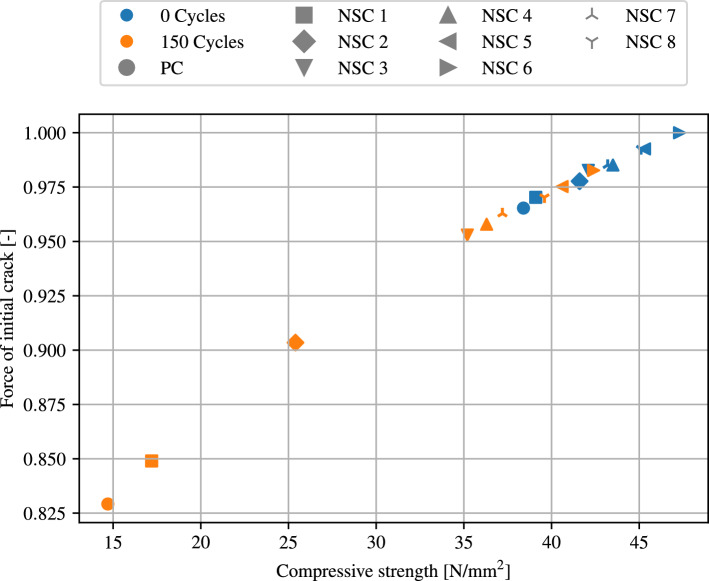
Evaluation of the force required for an initial crack for different compressive strengths before (blue) and after 150 (orange) freezing and thawing cycles. The different mixtures are denoted by different markers.

As a next steps, an evaluation of the force required to cause an initial crack for each composition of concrete with additional NS is performed. Figure 18 shows the force required for an initial crack at different compressive strengths, where the force is normalized to the maximal force. Normal concrete and 8 mixtures with different percentages of NS are evaluated, with values before the freezing and thawing cycle depicted in blue and values after 150 cycles in orange. The results show that the force required for crack initiation increases with increasing compressive strength. Since the specimens have a significantly lower compressive strength after the freezing and thawing process, crack initialisation start significantly earlier than in the initial states of the specimens. The graph also shows an evaluation of the individual mixtures. Here it is clear that the force required, increases with an increase in the NS percentage up to a value of 6% NS. This composition shows the highest resistance against cracking. Compounds with more than 6% NS, on the other hand, are less resistant.

## Conclusion

NS can enhance the microstructure of cement, thereby significantly enhancing the freezing resistance of concrete. In concrete containing NS, the microstructure and appearance of CSH gel are more uniform, compact, and arranged. Furthermore, NS results in fewer pores and tighter crystal coupling^83^. In our experimental and numerical studies, we found out, that:

- Adding NS caused a significant increase in compressive strength before and after freezing and thawing cycles. Samples showed an increase in compressive strength up to 6% NS. After this percentage, adding NS has a reverse effect on the compressive strength. The highest compressive strength of all samples was observed in NSC6, followed by NSC5 for different freezing and thawing cycles.
- When cement was replaced by NS ($\ge$ 4%), a marked reduction in water penetration depth was observed for concrete before and after freezing and thawing cycles. The best result was obtained by NSC8, giving a decrease of 57% in water permeability compared to the control sample after 150 freezing and thawing cycles.
- Cement mixes including NS decrease the chloride ion penetrability before and after freezing and thawing cycles. The charge passed was reduced by increasing the replacement. Very low permeability was obtained by the NS replacements of 4–8% to cement after freezing and thawing cycles and moderate permeability was recorded by samples with 2–3%. Among all samples, the highest chloride permeability reduction was noticed with NSC8 by 86%.
- Numerical simulations using phase-field theory has been used to capture crack propagation under loading in concrete by changing the compressive strength. This resulted in a reduction of the required force for crack initialisation of 13.88% for concrete with 6% NS compared to normal concrete.

Future research should cover an explicit description of freezing and thawing processes in concrete. Therefore, the multi-phasic nature of concrete could be modeled by describing concrete as a tri-phasic continua (concrete, water, air) based on a homogenization approach. Together with the incorporation of cracking mechanisms calibrated to specialized fracture experiments, enables a better understanding of the impact of freezing and thawing cycles on the lifespan of real-world constructions.

### Supplementary Information

Supplementary Information.

## Data Availability

Data underlying the results presented in this paper are not publicly available at this time but may be obtained from the corresponding author upon reasonable request.
