# Supplementary material for: On effects of freezing and thawing cycles of concrete containing nano-SiO2: experimental study of material properties and crack simulation
Source: Sci Rep. 2023 Dec 14;13:22278. doi: 10.1038/s41598-023-48211-4 (PMC10721871; doi:10.1038/s41598-023-48211-4)
Supplement: Supplementary file 1 — Supplementary Information. [file 41598_2023_48211_MOESM1_ESM.pdf]

# Supplementary Material for the manuscript: On the effect of freezing and thawing cycles of concrete containing nano-$SiO_2$: Experimental study of material properties and numerical investigation of crack propagation

O. Arasteh-Khoshbin, S. M. Seyedpour*, M. Brodbeck, L. Lambers, T. Ricken

Within this section validation results of the used phase-field model shall be discussed. The governing equations were discretised using Lagrangian finite-elements of first order and implemented into the finite-element framework FEAP[1]. For mesh generation the open-source software Gmsh[2] was used.

For the actual validation two test-cases, the so called tension-test[3] as well as the shear-test[4], were recalculated. Geometry as well as boundary conditions are shown in detail in Figure 1.

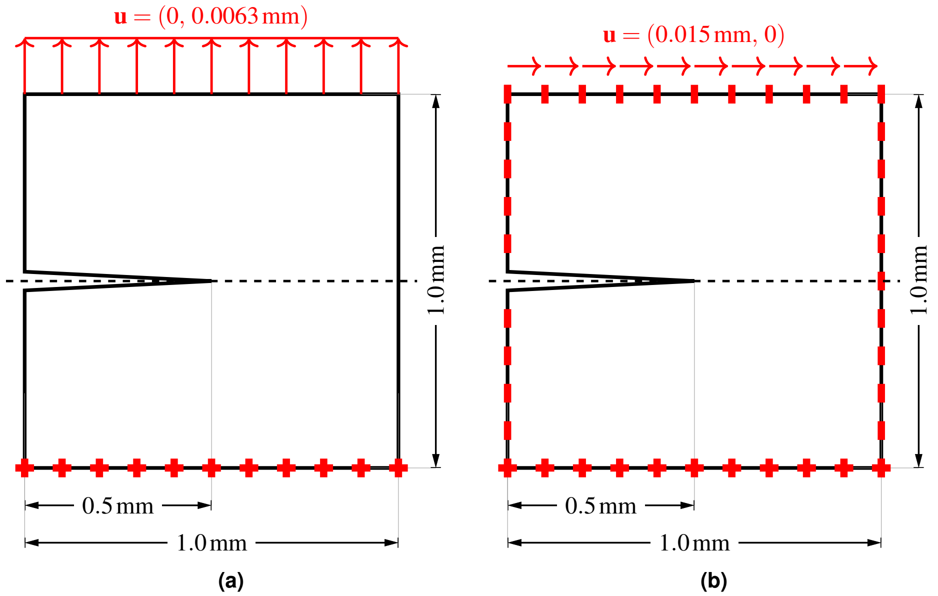


**Supplementary Figure 1.** Geometry and boundary conditions of (a) tension- and (b) shear test. The crack opening width at $x = 0$ is 0.001 mm. For the fracture phase-field Neumann boundary conditions are prescribed.

For both calculations the nonzero displacement boundary-condition on the top-surface is applied incrementally. The incremental displacements $\Delta u_i$ per pseudo-time-step is given in Table 2. To complete the description of the model, the utilised material- and model parameters are summarised in Table 1.

| | | | | | |
|---|---|---|---|---|---|
| $\lambda$ | $121.15\,\mathrm{kN\,mm^{-2}}$ | $\mu$ | $80.77\,\mathrm{kN\,mm^{-2}}$ | $g_c$ | $0.0027\,\mathrm{kN\,mm^{-1}}$ |
| $l_c$ | 0.015 mm | $k_c$ | 0 | $M_c$ | $0\,\mathrm{kg\,mm^{-1}}$ |

**Supplementary Table 1.** Material parameters used for the validation.

| | $n_{step}$ 1 – 500 | $n_{step}$ 501 – end |
|---|---|---|
| tension test | $\Delta u_2 = 1 \times 10^{-5}$ mm | $\Delta u_2 = 1 \times 10^{-6}$ mm |
| shear test | $\Delta u_1 = 1 \times 10^{-5}$ mm | $\Delta u_1 = 1 \times 10^{-5}$ mm |

**Supplementary Table 2.** Load ramp within the test-cases. $n_{step}$ denotes the number of time steps, where a load increment is applied.

As phase-field models are sensitive to the considered mesh. As Miehe et al.[3] did not publish mesh information, a unstructured, triangular mesh, locally refined in the crack region was used ($h_{min}$ within the crack-region). For the shear test - a test case, highly sensitive with respect to the mesh - Hesch and Weinberg[4] used a structured, quadrilateral mesh of $256 \times 256$ first order elements. The usage of an identical mesh – which should, assuming the correctness of the implementation, give equivalent results – make a comparison of the results easy.

The results of the here described test-cases are shown in Fig. 2 (a) for the tension- and in Fig. 2 (b) for the shear-test. Both results match the reference calculations quite well. The slight differences within the results of the tension test might either result from the fact, that Miehe at al. used a slightly different mesh or residual stiffness $k_c$. Both values were not reported within the paper.

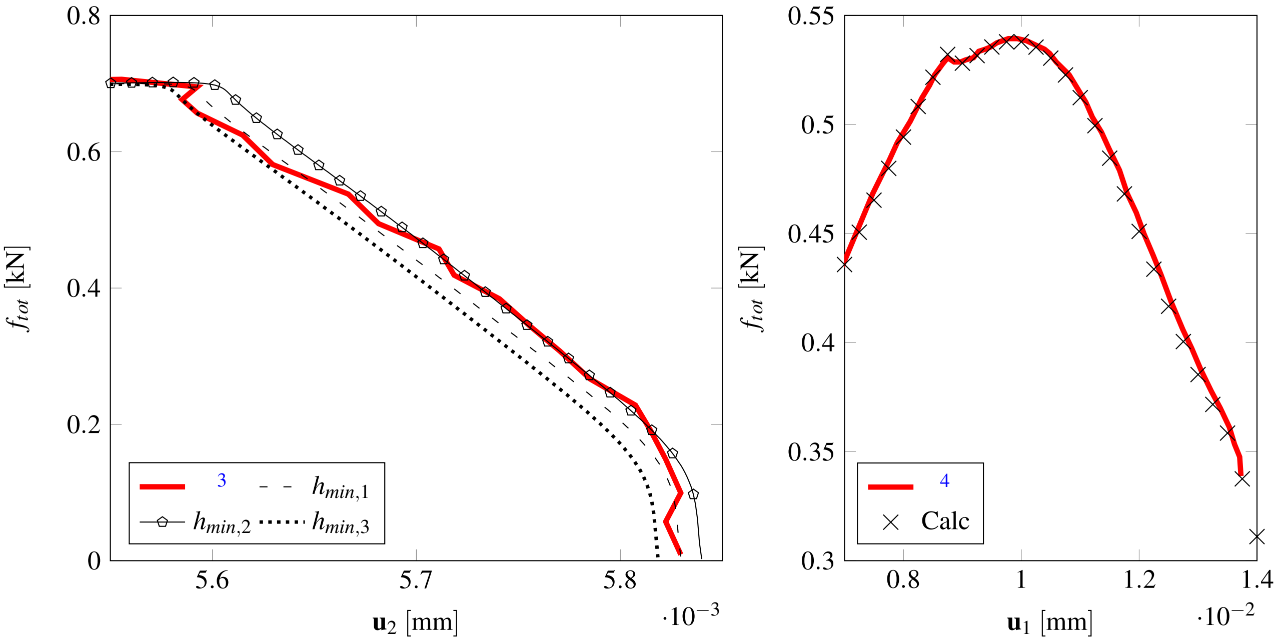


**(a)** **(b)**

**Supplementary Figure 2.** Force-displacement curves of (a) the tension- and (b) the shear test. Within (a) a sequence of meshes with minimal mesh sizes $h_{min,1} = 1 \times 10^{-3}$ mm, $h_{min,2} = 5 \times 10^{-4}$ mm and $h_{min,3} = 2.5 \times 10^{-4}$ mm is presented.

Further tests, which are not shown in the appendix, showed results within the expected scope. Therefore, the implementation can be seen as correct.

## References


**1.** Taylor, R. L. FEAP - finite element analysis program (2014).

**2.** Geuzaine, C. & Remacle, J.-F. Gmsh: A 3-d finite element mesh generator with built-in pre- and post-processing facilities. *Int. J. for Numer. Methods Eng.* **79**, 1309–1331 (2009).

**3.** Miehe, C., Hofacker, M. & Welschinger, F. A phase field model for rate-independent crack propagation: Robust algorithmic implementation based on operator splits. *Comput. Methods Appl. Mech. Eng.* **199**, 2765 – 2778 (2010).

**4.** Hesch, C. & Weinberg, K. Thermodynamically consistent algorithms for a finite-deformation phase-field approach to fracture. *Int. J. for Numer. Methods Eng.* **99**, 906–924 (2014).
